# Supplementary material for: A Critical View of the Application of the APEX Software (Aqueous Photochemistry of Environmentally-Occurring Xenobiotics) to Predict Photoreaction Kinetics in Surface Freshwaters
Source: Molecules. 2019 Dec 18;25(1):9. doi: 10.3390/molecules25010009 (PMC7017383; doi:10.3390/molecules25010009)
Supplement: Supplementary file 1 [file molecules-25-00009-s001.zip › 3.2.4_gcc-4.4.0.zip!/3.2.4_gcc-4.4.0/3.2.4_gcc-4.4.0/doc/octave/html/interpreter/Derivatives-and-Integrals.html]

Derivatives and Integrals - Untitled


Next: Polynomial Interpolation,
Previous: Products of Polynomials,
Up: Polynomial Manipulations


---

### 27.4 Derivatives and Integrals

Octave comes with functions for computing the derivative and the integral
of a polynomial. The functions `polyderiv` and `polyint`
both return new polynomials describing the result. As an example we'll
compute the definite integral of p(x) = x^2 + 1 from 0 to 3.

```
     c = [1, 0, 1];
     integral = polyint(c);
     area = polyval(integral, 3) - polyval(integral, 0)
     => 12
```

— Function File: **polyderiv** (c)  
— Function File: [q] = **polyderiv** (b, a)  
— Function File: [q, r] = **polyderiv** (b, a)  
> Return the coefficients of the derivative of the polynomial whose
> coefficients are given by vector c. If a pair of polynomials
> is given b and a, the derivative of the product is
> returned in q, or the quotient numerator in q and the
> quotient denominator in r.
>
>
>
> **See also:** poly, polyinteg, polyreduce, roots, conv, deconv, residue, filter, polygcd, polyval, polyvalm.

— Function File: **polyder** (c)  
— Function File: [q] = **polyder** (b, a)  
— Function File: [q, r] = **polyder** (b, a)  
> See polyderiv.

— Function File: **polyinteg** (c)  
> Return the coefficients of the integral of the polynomial whose
> coefficients are represented by the vector c.
>
> The constant of integration is set to zero.
>
>
>
> **See also:** polyint, poly, polyderiv, polyreduce, roots, conv, deconv, residue, filter, polyval, polyvalm.

— Function File: **polyint** (c, k)  
> Return the coefficients of the integral of the polynomial whose
> coefficients are represented by the vector c. The variable
> k is the constant of integration, which by default is set to zero.
>
>
>
> **See also:** poly, polyderiv, polyreduce, roots, conv, deconv, residue, filter, polyval, polyvalm.
